# Exploring physical symptoms and distress in early‐stage breast cancer survivors on hormone therapy: A qualitative study

**DOI:** 10.1111/bjhp.70012

**Published:** 2025-08-18

**Authors:** Sophie Fawson, Zoe Moon, Melanie Rattue, Rona Moss‐Morris, Lyndsay D. Hughes

**Affiliations:** ^1^ Health Psychology Section King's College London London UK; ^2^ National Institute for Health and Care Research, Maudsley Biomedical Research Centre London UK; ^3^ Faculty of Life Sciences UCL School of Pharmacy, University College London London UK

**Keywords:** breast cancer, distress, qualitative, symptoms

## Abstract

**Objectives:**

Distress and physical symptoms are ongoing for early‐stage breast cancer survivors on adjuvant hormone therapy. While previous qualitative research has focused on reporting the outcomes of distress and symptoms, as well as medication adherence, this study aimed to understand the link between symptoms and distress, exploring specifically how symptoms are distressing to inform interventions.

**Design and Methods:**

A qualitative design was used with online semi‐structured interviews conducted with 23 women with Stage I–III hormone receptor positive breast cancer, living in the United Kingdom and prescribed hormone therapy in the previous 2 years, about their experience of hormone therapy. Interviews were transcribed verbatim and analysed using inductive reflexive thematic analysis.

**Results:**

The emotional burden of side‐effects was an overarching theme. Other themes depicting why symptoms are distressing included the sense of helplessness around symptoms, living with and managing difficult feelings around loss and change, living with fear, worry and uncertainty around treatment side‐effects and the internal conflict when making treatment decisions.

**Conclusions:**

The findings indicate specific areas of emotional need in women prescribed hormone therapy related to living with and managing physical symptoms. This provides insight into how symptoms can contribute to distress, which goes beyond previous research that reports the perceived consequences of symptoms and distress. The results, therefore, can inform healthcare professional communication to support and validate ongoing experiences and the underlying decision conflict, which may be distressing for some women. Furthermore, psychological interventions could address the sense of loss and acceptance of limitations and change.

**Statement of contribution**

**What is already known on this subject?**

Previous qualitative and observational studies have identified that physical symptoms are distressing for early‐stage breast cancer survivors on hormone therapy and that both symptoms and distress contribute to outcomes such as poor quality of life and medication non‐adherence, increasing the risk of cancer recurrence and death. However, less is known about *how* and *why* these symptoms are distressing. Understanding the psychological and emotional burden of managing physical symptoms may help to develop more targeted and effective interventions.

**What does this study add?**

- The study provides an in‐depth understanding of *why* hormone therapy side effects are distressing.
- Psychological approaches may be useful for acceptance and managing the sense of loss experienced.
- Clinical communication should include clear side effect expectations, validation and empathy.

## INTRODUCTION

Breast cancer survivors prescribed hormone therapy such as tamoxifen or aromatase inhibitors (AIs) not only have to manage general survivorship burdens such as the impact of frequent medical contact ending, uncertainty around the future and fears of cancer recurrence and ongoing physical and psychological symptoms (Burgess et al., 2005; Lethborg et al., 2000; Schreier et al., 2019; van den Beuken‐van Everdingen et al., 2008), but also have to take a daily medication that comes with additional challenges. The effect of hormone therapy on the reduction or interference of oestrogen can have significant physical and psychological effects (Hunter et al., 2004) including joint pain and stiffness, fatigue, headaches and menopausal symptoms such as hot flushes, night sweats and vaginal dryness (Garreau et al., 2006; Rademaker et al., 2025).

Distress is prevalent in breast cancer survivors and is associated with poor health outcomes such as lower quality of life, non‐adherence to hormone therapy, and increased personal and healthcare costs (DiMatteo & Haskard‐Zolnierek, 2011; Fang & Schnoll, 2002; Moon et al., 2019; Phoosuwan & Lundberg, 2022; Waller et al., 2013). Symptoms such as pain, menopausal symptoms and fatigue have been found to be associated with distress (Andreu et al., 2022; Lambert et al., 2018; Moon et al., 2016; Syrowatka et al., 2017), observational studies are limited in their ability to explore the underlying reasons for and the nuances of distress.

Previous qualitative studies have corroborated that symptoms are distressing (Harrow et al., 2014; Ibrar et al., 2022; Jacobs et al., 2020; Wen et al., 2017). A thematic synthesis of 16 qualitative studies identified themes related to the daily impact of side effects, the role of healthcare professionals in preparing women for side effects and supporting adherence, strategies to manage side effects and the impact on adherence and weighing up pros and cons of taking hormone therapy (Peddie et al., 2021).

Although revealing important consequences of symptoms, previous research has failed to provide insight into *how* and *why* symptoms are distressing and focuses heavily on the impact on treatment adherence (Jacobs et al., 2020). However, as women report persevering with their medication despite difficult symptoms (Lambert et al., 2018), it is important to recognize distress as an outcome regardless of its relation with behavioural outcomes, particularly as increased distress is related to lower quality of life (Moon et al., 2016) and increased healthcare utilization (Waller et al., 2013). As symptoms related to hormone therapy persist (Fawson, 2024; Moon et al., 2016), identifying *why* and *how* symptoms are distressing could identify targets for future interventions to help manage distress alongside encouraging long‐term adherence.

Qualitative research provides a framework to explore the experiences of breast cancer survivorship which can help to provide context and uncover unknown mechanisms through which to understand theorized contributors to distress and identify the process through which symptoms are deemed distressing. Therefore, the overall aim of the present study was to qualitatively explore the distress breast cancer survivors on hormone therapy experience with the following research question: why are physical symptoms/side‐effects distressing for early‐stage HR+ breast cancer survivors?

## METHODS

### Study design and data collection

Semi‐structured interviews with open questions were used to allow for an open discussion exploring experiences of distress in women on hormone therapy. Due to the COVID‐19 pandemic, recruitment and interviews were conducted online through video conferencing software (Microsoft Teams). A critical realist approach was adopted as this aims to report on the experiences of the participants as real and true to them, not as a direct reflection but interpreted and acknowledged to inform further understanding of the data (Willig, 2013). A sample of 20–30 was estimated a priori to be adequate using general standards of qualitative interview analysis (Sim et al., 2018). Using the concept of information power (Malterud et al., 2016) a sample size on the lower‐to‐mid end of the original estimation was determined to be sufficient as although the research question was broad and an inductive approach was taken, the population was specific and the researchers were experienced in qualitative methods. With non‐response in mind, a target of approx. 30 was recruited to result in a suitable sample size.

### Participants and procedure

Breast cancer survivors were eligible for the study if they were over 18, lived in the UK, had a diagnosis of female Stage I–III breast cancer and had been prescribed hormone therapy in the last 2 years. Ethical approval was granted by King's College London Psychiatry, Nursing and Midwifery Research Ethics Committee HR‐19/20‐18770 and informed consent was obtained before commencing. The study was advertised through social media (e.g., Facebook, Instagram, Twitter), the university research recruitment circular and relevant charities. After consenting, eligible participants completed a short demographic and clinical questionnaire for purposive sampling. Thirty‐two eligible women were approached to arrange an interview with author SF or MR. Eight women did not respond to the email, and one had a delay in starting hormone therapy, so therefore was not eligible, leaving a sample of 23 women. Informed consent was confirmed over Teams and participants were fully debriefed. Interviews were conducted between October 2020 and July 2021. The interview schedule was reviewed by a patient advisory group as well as an experienced clinical psychologist (see supporting information for interview schedule). Interviews were conducted independently by two interviewers, recorded, securely stored, transcribed verbatim using the Microsoft Teams software and checked for accuracy. The mean duration of interviews was just under 1 h (57 min, SD 14, range 30–89 min).

### Data analysis

The interviews were analysed using inductive reflexive thematic analysis (Braun & Clarke, 2006; Braun & Clarke, 2019). Reflexive thematic analysis is a theoretically flexible approach and emphasizes the researcher as active in the analysis of the data (Braun & Clarke, 2019). This approach allows an in‐depth exploration of the interviewee's experiences and perceptions from a data‐driven perspective rather than having a predefined theory or framework and acknowledges the interplay between the data, the researcher, the research question and the environment and therefore particularly suits an open purpose (Braun & Clarke, 2023). The researcher's influence when interpreting the data is considered in the reflexive report (see Supporting Information).

Braun and Clarke's six stages for reflexive thematic analysis were followed (see Table 1, Braun & Clarke, 2006, 2019). The process was iterative during data collection; moving between coding and theme generation (Braun & Clarke, 2019). NVivo 12 (QSR International Pty Ltd., 2018) was used to store and organize codes.

**TABLE 1:** Phases of reflexive thematic analysis.

Phase of analysis	Details from this study
Familiarizing with the data	Author SF listened to all audio recordings, checked transcripts and made initial familiarization notes
Generating initial codes	Using NVivo 12 (QSR International Pty Ltd., 2018) author SF generated initial codes based on semantic explicit meaning A second round of coding was completed grouping and combining codes and creating more latent, interpretive codes The codes were discussed with the other authors and in line with the reflexive thematic analysis approach, the research question was refined
Generating initial themes	Initial themes and subthemes were generated based on overarching concepts, shared meaning and patterns in data, rather than topics (Braun & Clarke, 2019)
Reviewing themes	Themes were then reviewed and discussed with the authors, who all have background knowledge of this area, and developed and refined multiple times using multiple thematic maps before naming final themes and subthemes
Defining and naming themes	
Producing the report	Relevant quotes chosen and analysis related back to the research question and literature

Some of the included quotes were edited to remove repetitions and […] indicates a skip to later content, when meaning and content is congruent. In terms of quality assessment, in line with guidance for reflexive thematic analysis, themes were conceptualized as containing shared meaning rather than shared topics, developed through the researchers' involvement and codes were developed through ongoing interpretation and involvement with the data. Although Braun and Clarke (Braun & Clarke, 2023) recommend best practice for thematic analysis away from standardized measures, this paper reports in line with their best practice guidelines as well as generic Standards for Reporting Qualitative Research (SQRQ; O'Brien et al., 2014).

### Techniques to enhance trustworthiness

As highlighted in the SQRQ guidelines (O'Brien et al., 2014) to ensure credibility and reliability in the study findings, an audit trail was kept with a coding manual reviewed by the authors with frequent discussion and the coder did not experience breast cancer allowing distance and full immersion in the data with fewer preconceived ideas. The full reflexivity statement is in Supporting Information.

## RESULTS

Twenty‐three women with a mean age of 53 (SD 11) were interviewed. See Table 2 for aggregate demographic and clinical characteristics. The mean time women were on hormone therapy was 9 months (SD 7). Seventy‐eight per cent were White British, with an even split between tamoxifen and AIs. For context, physical symptoms experienced are displayed in the supporting information (Table S3). Symptoms were perceived to be side‐effects of hormone therapy, so the terms have been used interchangeably throughout.

**TABLE 2:** Participant demographic and clinical characteristics.

	Aggregate *n* = 23
Age (*M*, SD, range)	52.57 (10.94; 33–81)
Ethnicity (*n*)	
White British	18
Other White background	3
Black Caribbean	1
Indian	1
Marital status (*n*)	
Married or in a civil partnership	17
Single or co‐habiting	4
Separated/divorced	1
Widowed	1
Stage at diagnosis (*n*)	
Stage I	10
Stage II	8
Stage III	5
Current hormone therapy (*n*)	
Tamoxifen	12
*Aromatase inhibitors*	
Letrozole	5
Exemestane	2
Anastrozole	4
Months on hormone therapy (M, SD, range)	9.34 (7.45; 0.25–27)

*Note*: *M*, mean; SD, standard deviation.

**FIGURE 1:**
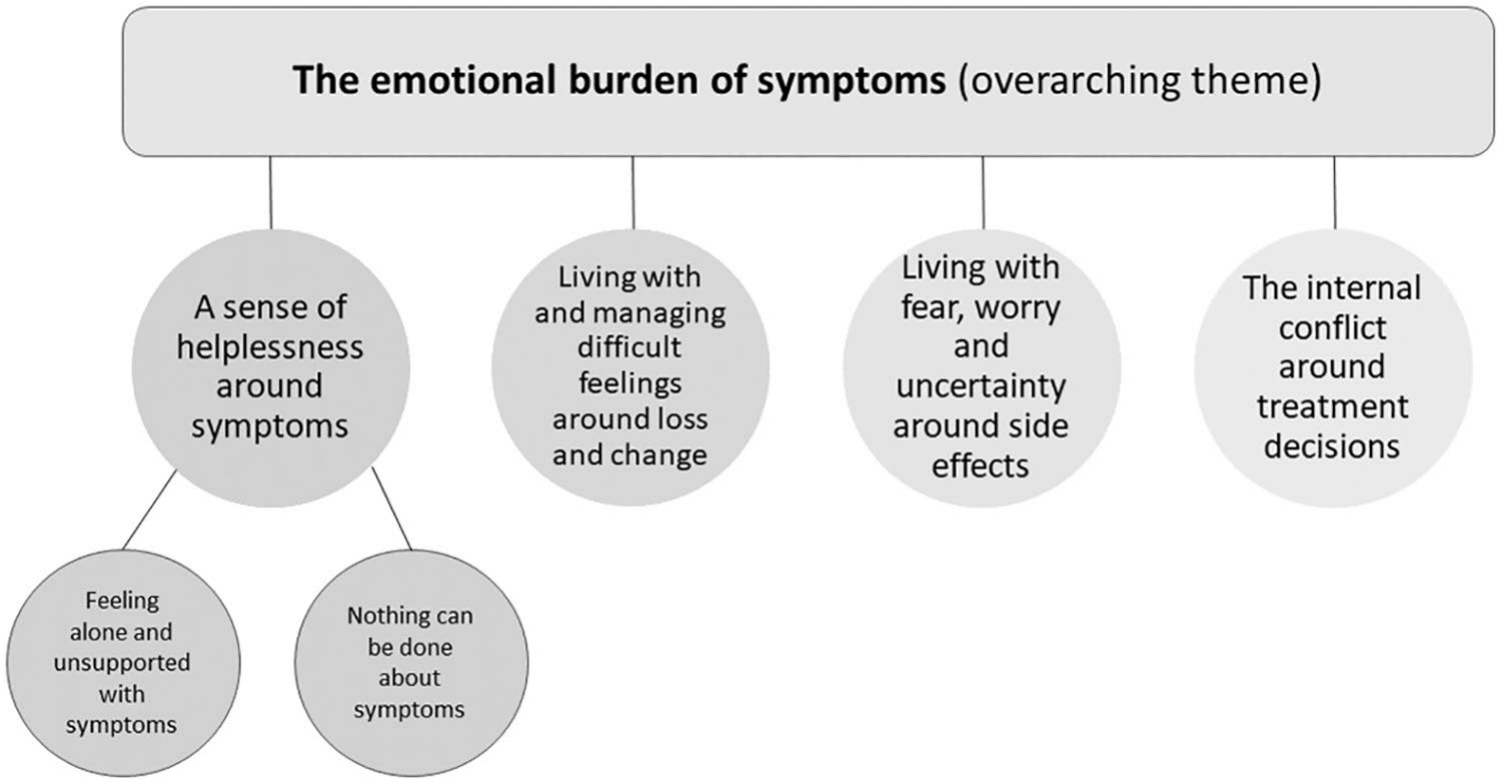
Thematic map.

Figure 1 displays the four themes and two subthemes generated from the data. Emotions were expressed in conjunction with symptom experience throughout, which is highlighted as an overarching theme; *the emotional burden of symptoms*. However, experiences went beyond simply identifying the emotional experience of symptoms alone, and the remaining themes detail why symptoms are distressing for women on hormone therapy. These themes will be discussed with participant quotes (additional quotes for themes are available in Supporting Information).

### Overarching theme: The emotional burden of symptoms

The supporting information provides quotes to demonstrate and give context to the range of emotions and emotional language used to specifically describe symptoms experienced while on hormone therapy, including low mood, frustration and anger. These were described by most women regardless of their time on or type of therapy.

Some women said symptoms were ‘manageable’ and not ‘terribly difficult’ (P22, [age] 49, tamoxifen). However one described that ‘the distress comes from the cumulative effect of having a lot of little things’ (P16, 47, exemestane). Older age appeared to be a possible protective factor for distress around menopausal symptoms such as hot flushes and vaginal dryness.

#### Theme 1: A sense of helplessness around symptoms

There was a sense of helplessness around symptoms whereby not only did women feel a lack of understanding and support around symptoms, but also felt that nothing could be done about them.

##### Sub theme 1.1: Feeling alone and unsupported with symptoms

Women described a heightened sense of feeling alone and unsupported after primary treatment as they are being ‘left with this box of tamoxifen’ which was ‘more distressing’ (P5, 39, tamoxifen).

Women felt invalidated and ‘discounted’ when oncologists seemed to ‘shrug’ off their concerns (P8, 49, tamoxifen) or attributed symptoms to other causes which left women feeling they have a lot to learn themselves, and that the impact is not understood. On the other hand, women who felt listened to and were told that side effects and symptoms were normal, appreciated the validation, reassurance and knowledge. This reassurance came from different sources such as breast care nurses, social support online groups and personal research. One participant explained how breast cancer nurses reassured them that side effects are normal, giving them ‘the ability to kind of go OK, it is what it is’ (P22, 49, tamoxifen). Another participant explained how seeing others' experiences through Facebook was reassuring ‘to know that other people are going through the same thing’ (P14, 45, anastrozole and goserelin).

Women also described feeling unsupported by family and friends. Women felt that because their difficult side effects were not visible, there was a lack of understanding from loved ones and others. One woman described how her ‘family laughs at me because they think I'm making it up’ (P12, 49, letrozole and goserelin) suggesting support and understanding may be limited.

And I just thinking I'm not OK though, I don't feel OK, I know I look OK but I don't feel OK. (P15, 51, tamoxifen)

Due to the COVID‐19 pandemic, some face‐to‐face support and appointments were withdrawn, and women found it ‘difficult to ask for things over the phone’ (P14, 45, anastrozole and goserelin) leaving some wondering if the pandemic limited their access to beneficial support. However, it seems that even those participants whose treatment started before the COVID‐19 pandemic felt unsupported and alone and therefore this does not appear to be specific to the COVID‐19 pandemic changes.

##### Sub theme 1.2: Nothing can be done about symptoms

There was a sense of helplessness that nothing can be done about side effects or symptoms; therefore, women just had to deal with, live with or suffer with them.

There's not a lot of help out there, you just kind of have to suffer it really. That's the only way. It's a shame. (P19, 43, tamoxifen and goserelin)

Although some women felt there was medication available to help manage symptoms and side effects, they were aware of the contraindications, and some were reluctant to take further medication on top of hormone therapy medication. While one participant felt you cannot actually make symptoms better or worse, rather things improve by learning to cope.

Some women felt they had no option but to be on hormone therapy itself as it is the ‘blue ribbon treatment’ (P11, 53, anastrozole then letrozole). For some, there was a desire for it to ‘be a choice rather than a necessity’ (P3, 33, tamoxifen) as those who felt they had a choice or option to stop taking hormone therapy for a period of time or change medication, felt having that sense of control helped their distress around symptoms.

Just knowing that that option was there [to stop Letrozole], I think in some ways gave me something to focus on and to make a decision about rather than feeling hopeless. (P12, 49, letrozole and goserelin)

#### Theme 2: Living with and managing difficult feelings around loss and change

Difficult symptoms were described in conjunction with limitations to lifestyle and losses. Women used strong emotional language to describe ‘not being able to do the things I could before’ and dealing with ‘loss’ to parts of their lives (P14, 45, anastrozole and goserelin).

And no you can't pick that up when you can't do this hobby and duh duh duh. It's death by all of these things. (P16, 47, exemestane)

A specific area of loss which was unexpected and difficult to manage, was related to vaginal dryness and the impact of this on their sex lives. This ‘loss’ (P18, 48, tamoxifen) of an important area of their lives was something they never thought they would have to deal with and ‘had no idea of the impact’ (P23, 49, letrozole).

In addition, women reported challenging disruptions to their sense of self while taking hormone therapy. Women described how they now felt like someone with health issues on medication when they hadn't ever taken medication before. Women referred to the physical impact of pain meaning they feel they have ‘aged about 20 years’ (P20, 75, anastrozole then tamoxifen). Women felt too young to be going through the menopause and this was described in conjunction with feelings of sadness and low mood.

I feel too young to be having them [menopausal symptoms], so it does, it does get me down quite a lot. (P19, 43, tamoxifen and goserelin)

Additionally, women commented on how these changes ‘zaps some vitality for life out of you’ (P11, 53, anastrozole then letrozole) and felt that their personality and identity was being ‘chipped’ away.

You know that your sense of self and your identity is just. It's just being chipped at you know it's had a couple of great big fucking knocks taken out of it and then it's like oh we're just gonna chipping and keep chipping. (P16, 47, exemestane)

Women reported feelings of uncertainty around whether they would ever feel normal again. One respondent disliked the term ‘new normal’ that is often used after primary treatment and felt that it being described in this way implies ‘you'll get back to your normal, it'll just feel different’ whereas it is actually ‘abnormal’ as you ‘don't ever really get a chance to feel like yourself again’ (P16, 47, exemestane).

Some women reported that life wasn't ‘awful’ but ‘that comes down to adapting’ (P23, 49, letrozole) to these changes and dealing with this loss. However, learning to adapt and live differently can take time especially as some women felt the limitations made their body feel ‘less resilient’ which then made it harder to cope with change (P11, 53, anastrozole then letrozole).

It's [physical problems] kind of there all the time so you learn to live differently. (P21, 54, anastrozole)

Managing and coping with these difficulties was done in different ways. Firstly, practical strategies were used such as ‘managing my diary very carefully and I absolutely have to factor in rest time […] to just sort of function and cope’ (P23, 49, letrozole). However, accessing practical aids to manage and adapt to symptoms such as vaginal lubricants was felt to be ‘embarrassing’ (P19, 43, tamoxifen and goserelin). One woman used practical devices to manage the impact of her symptoms however she described this as a frustrating and irritating thing to have to do, despite helping her manage:

As irritating as it is, I think actually some practical aids to just help take some of the difficulty out of life, I think that does help. (P16, 47, exemestane)

Another strategy was a more psychological response, whereby this same participant described having to complete self‐talk to not be so ‘vain’, and accept help while losing attachment to what they used to have.

There is a sense in which you lose attachment to what you used to have and there is that gradual acceptance of the fact that OK, do you know what you're, you are below par and you may never get back to the par you had, but you can adapt and you can adjust. (P16, 47, exemestane)

Adapting and adjusting was described as doing ‘things differently rather than focusing on what I can't do’ (P14, 45, anastrozole and goserelin) for example, ‘if you can't do your exact hobby, find a near enough or a good enough alternative’ (P16, 47, exemestane).

However, another participant was aware they could not accept their limitations:

I think I haven't quite accepted my limitations, and I'm not sure that I ever will if I'm honest. (P19, 43, tamoxifen and goserelin)

#### Theme 3: Living with fear, worry and uncertainty around side‐effects

Women reported a range of unknown and unexpected aspects related to being on hormone therapy, which resulted in fear, worry and uncertainty. Some women reported being very ‘apprehensive’ (P5, 39, tamoxifen) and ‘wary’ (P13, 51, tamoxifen) when first prescribed hormone therapy, particularly about potential side‐effects. In addition, women initially thought side‐effects would be manageable, so the actual impact of the distressing symptoms was unexpected.

One woman mentioned physical symptoms persisting ‘for longer than I realized that they would’ (P22, 49, tamoxifen) and other women felt the thought of experiencing side‐effects for a long time was unnerving, frustrating and worrying, with some women worrying the side‐effects could actually get worse, and continue to negatively impact their lives.

Letrozole for 10 years and it was just seemed I just didn't think I was going to be able to cope with it. It's just unbearable. (P12, 49, letrozole and goserelin)

It seems that joint pain might be an issue later on, and that's something which I'm quite worried about because of again, that's going to stop me doing the things I want to do and being active and everything, yeah (sighs). (P3, 33, tamoxifen)

Another element of uncertainty was women questioning whether they would have experienced similar symptoms of a normal menopause.

I mean what's hard to know is if I'd gone through the menopause, would I just be experiencing very similar things to this and is it just that I'm going through an accelerated menopause? (P1, 46, tamoxifen)

One participant described the experience of symptoms leading to worries and concerns of future medical conditions.

I remembered almost worrying and thinking, oh my gosh, could I get dementia from this drug because of the, you know the brain fog and the concentration being able to concentrate. Yeah, those things are distressing and worrying. (P8, 49, tamoxifen)

Finally, some women reported fears around changing their hormone therapy and having worse side‐effects on an alternative medication. When suggested she could change from tamoxifen to AI's to manage bone pain side‐effects, one participant expressed their concern that they could experience ‘worse bone pain’ and felt it would not be ‘a very good idea’ (P1, 46, tamoxifen).

Despite the uncertainty, some women were hopeful that things might improve, especially those who had only recently started taking their medication.

But I'm hoping I'm just going to get them for a few months with any luck. (P9, 63, anastrozole)

#### Theme 4: The internal conflict around treatment decisions

Women reported an internal conflict as they weighed up whether taking hormone therapy was the right decision for them. This included the conflict between quantity versus quality of life, which women related to the experience of the difficult side‐effects versus the reduced risk of recurrence.

I'm still young. I still got children. I've got everything to live for. And yet, it's a very difficult decision to make. Do I carry on with these horrific drugs and the side‐effects are indescribable sometimes? Or do I say you know what I want to have quality of life, but at 54, I need both. I don't just want quality of life […]. (P11, 53, letrozole)

This internal conflict also includes struggles with questioning decisions to switch between AIs and tamoxifen. One participant's oncologist suggested changing medication which left them weighing up different symptoms over others and the potential increase in risk of recurrence. Some women felt that they did not have the knowledge or information to make an informed personal decision about being on hormone therapy which contributed to this internal conflict.

Questioning decisions to stay on hormone therapy was ongoing, with some women reporting a taste of normality and improved side‐effects when taking a break from their medication. However, this added to the difficulties with decision making, with women stating that hearing others miss tablets and taking ‘tamoxifen holidays’ made them question taking their medication as they were ‘starting to feel normal again’ (P4, 57, letrozole, exemestane then tamoxifen).

Although these women reported struggling with these conflicts and decisions, on the other hand, other women reported a more positive focus and seemed to experience less struggle and conflict. They thought of hormone therapy as ‘a pretty good alternative’ (P18, 48, tamoxifen) to a recurrence or even ‘better than dying’ (P12, 49, letrozole and goserelin). They felt it gives them ‘less chance of it [cancer] returning’ *(P3, 33, tamoxifen)* and felt this was a good thing that gives them hope. Women felt managing side effects were therefore a ‘small price to pay’ (P19, 43, tamoxifen, Goserelin). Women reported that taking hormone therapy and going through treatment ‘affords you a life to live’ and means they are around for family and ‘still here as a friend’ (P2, 63, letrozole). However, women used quite strong terminology to describe coping with and managing this internal conflict. One participant said, ‘I'm trying my hardest to accept’ (P3, 33, tamoxifen) to imply that this shift of focus or reframing was a cognitive effort to try and think about taking hormone therapy and symptoms in a different way. A sense of self‐imposed pressure and a forceful nature was also interpreted from the data.

You have to be quite strong‐minded (P11, 53, letrozole)

I just think well suck it up it's, it's better than the alternative. (P19, 43, tamoxifen and Goserelin)

## DISCUSSION

The current study provides a detailed insight into the distress experienced while on hormone therapy by early‐stage breast cancer survivors. In line with previous literature (e.g., Rosedale & Fu, 2010), the majority of women described the emotional burden and distressing nature of symptoms and side‐effects themselves, which was the overarching theme throughout this study. However, the current analysis went beyond previous literature by describing the in‐depth emotional impact of the side‐effects associated with being on hormone therapy and exploring the nuances of *why* physical symptoms are distressing for these women. The study has identified that it is not just the presence of the symptoms, but the impact, burden and disruption to daily lives of these symptoms that may lead to distress.

The first theme highlighted a strong sense that some women feel they have been left alone to self‐manage hormone therapy and the additional experience of side‐effects, with limited support and knowledge from health care professionals. This study found that feeling unsupported left some women feeling helpless and felt the lack of information made them question whether their experience was normal and felt unheard, not understood and therefore invalidated for their experiences. Although previous literature links feeling unsupported by healthcare professionals to decisions to stop taking medication (Peddie et al., 2021), this present study highlights that these experiences may also lead to distress as well, outside of the decision to take medication or not. Interestingly, individuals who felt a sense of control over changing their medication reported that this helped them to cope with their situation. This may help mitigate the experience of symptoms leading to helplessness and resultant distress. However, some women reported fears and concerns around unknown side‐effects from a different medication. Clear expectations and plans for managing new potential side‐effects should be discussed to avoid women settling for an unsatisfactory but familiar treatment experience to avoid potential unfamiliar side‐effects. Moreover, individuals felt limited support and understanding from their family, friends or loved ones which may be partly due to the invisibility of symptoms. Women often reported finding support for managing symptoms and distress through social media and from others with similar experiences, which helped with validating and normalizing experiences.

There was also a sense of helplessness around managing symptoms. Previous literature has highlighted a lack of self‐management options for hormone therapy side‐effects (Hall et al., 2022); however, the women in this study felt helpless and like they had to suffer. This further justifies the exploration of why symptoms might lead to distress. Rather than directly targeting management of symptoms, which often has limited success, interventions could target self‐efficacy in managing symptoms (Hoffman, 2013) or coping with the experience of symptoms, which might be beneficial for mental health outcomes by alleviating some of the distress expressed about lack of control and helplessness.

A separate theme describes uncertainty and worry around symptoms continuing throughout survivorship with regards to side‐effects worsening and persisting for longer than expected, which they felt could have longer detrimental health impacts. Feeling unprepared and experiencing surprising side‐effects has been reported previously as influencing an individual's decision to stop taking their medication (Peddie et al., 2021). However, this current study highlights the ongoing nature of these feelings, with an emphasis on the fear of the unknown over the course of the prescribed treatment period. This may link to the feeling of being unsupported, as some of these expectations and information giving could be provided in a more accessible way to relieve these concerns.

This current study highlights specific psychological and emotional impact from experiencing symptoms whereby women reported feelings of loss about their sense of self changing, and feeling older than they should because of pain and stiffness. Although these symptoms could themselves be a sign of aging, these women attributed them to AET. This is important because this may amplify feelings of unfairness and lack of control, and in this study, women often reported feeling helpless and resigning to these outcomes. Others reported frustration and embarrassment with the physical changes and having to adapt to them. Accepting limitations or persisting with difficulties was challenging as some could not see past their limitations. In previous literature, women have reported struggling with reintegrating into their pre‐cancer lives (Costanzo et al., 2007), and in this study, women also identified that the often‐public narrative around the ‘new normal’ was not helpful as the continued physical and psychological changes from continuing hormone therapy represented a more permanent change from before diagnosis.

Breast cancer survivors weigh up the pros (reduction in risk of recurrence) and cons (side‐effects impacting quality of life) of taking hormone therapy, and this has usually been investigated in relation to the outcome of treatment adherence (Ibrar et al., 2022; Moon et al., 2017). However, this study highlighted that the decision‐making process is a burden and an ongoing source of distress and internal conflict. Those who tried to shift to a more positive stance about taking hormone therapy described this as requiring cognitive effort with a sense of self‐imposed pressure to take the medication and having to endure symptoms to be around for their family. This feeling of obligation, also found in Peddie et al. (2021), could be a challenging concept to reconcile while being adherent. Support to accept the decision they have made may help manage the ongoing difficult thoughts about the process.

Although not a specific aim of the qualitative study, there seemed to be no differences between the distress experienced from those taking AIs compared to those on tamoxifen, with a range of participant quotes contributing to the themes. This distinction is often not explored in qualitative research (e.g., Clancy et al., 2020; Lambert et al., 2018; Peddie et al., 2021), but quantitative research suggests that there are no differences in the magnitude of emotional distress between the two types (Ates et al., 2016). This is despite research suggesting that menopausal symptoms are more common for those on tamoxifen, while joint pain is more common with AIs, due to the different medications' impact on oestrogen (Garreau et al., 2006; Morales et al., 2004). If experiences are common between those on tamoxifen and AIs, this provides opportunities to inform clinical practice and interventions to treat women on either medication. This would be particularly useful as switching between the different types of hormone therapy is common whether due to side effects or menopausal status change (Kwan et al., 2017), meaning a potential intervention would be transferable.

Overall, the themes presented provide an indication of *why* symptoms are distressing for this population of breast cancer survivors. The participants described physical symptoms in relation to them feeling unhappy, sad and distressed, rather than in relation to stopping medication. Focusing on the facilitators and barriers to adherence as the existing body of research has done may miss this important outcome, particularly in those who persist with medication, continue to experience side‐effects and therefore distress. Furthermore, those who stop taking medication may still experience physical symptoms from previous treatment as well as the ongoing conflict about their decision to discontinue the medication. Therefore, this study has provided a unique focus towards the outcome of distress and how physical symptoms, or side‐effects of hormone therapy, may lead to distress. Highlighting distress as a meaningful outcome in this population is important, given its potential cost‐related and personal implications (Fang & Schnoll, 2002; Waller et al., 2013).

### Clinical implications and future directions

The current study supports the need for improved communication and expectation setting at hormone therapy initiation (e.g., Clancy et al., 2020; Lambert et al., 2018) and extends our understanding of ongoing challenges of accepting and managing side effects. An empathetic approach with validation, acknowledgement and normalizing experiences would address the sense of helplessness and abandonment, ongoing decision burden and uncertainty with symptoms, ensuring information is conveyed meaningfully, rather than just providing more information about symptoms. It has been previously reported in the literature that poor healthcare interactions can lead to worse outcomes, particularly in women from minority ethnic backgrounds (Moon et al., 2020; Tompkins et al., 2016) and that trust in the healthcare professional is associated with better outcomes (e.g., Birkhäuer et al., 2017), so the impact of these interactions on symptom experience and distress should be tested in future research.

Although patient‐initiated follow‐up is widely implemented, self‐management support interventions that correctly target the identified sources of emotional difficulties could supplement or substitute direct clinician support. Digital self‐management interventions in particular have the potential to be cost‐effective, accessible and tailorable to participants' needs (Ebert et al., 2018; Krebs et al., 2010). Although an inductive approach was taken, the themes can map onto cognitive behavioural treatment approaches that may be beneficial for altering or reframing any difficult thoughts around potential unknown challenges and improving understanding of treatment. Alternatively, a more third wave cognitive behavioural approach such as acceptance and commitment therapy (ACT; Hayes et al., 2006), promoting more flexible, accepting and compassionate responses may be more appropriate for the experiences around losing attachment to a previous sense of self and accepting loss of ability and physical limitations and decisions to be on hormone therapy. Addressing these factors could improve emotional burden, decrease distress and therefore improve quality of life, as has been found in other cancer samples and in long‐term conditions (Graham et al., 2016). Treating distress also has the potential to improve medication adherence, reduce cancer recurrence and reduce healthcare costs (DiMatteo & Haskard‐Zolnierek, 2011; Early Breast Cancer Trialists' Collaborative Group, 2011; Moon et al., 2019; Waller et al., 2013).

Different patient experiences have implications for future support. Stage of life was a potential protective factor as older women mentioned being less emotionally distressed, particularly by menopausal symptoms. Younger women may need increased support to manage symptoms and their associated impacts such as reduced vitality, sexual difficulties and stiffness. As there was no clear pattern of differences between the emotional experiences of women on tamoxifen and AIs, it may be that the collective experience of managing the helplessness, change and loss linked to the symptoms and the ongoing decision‐making is where the distress is coming from. Future communication and interventions could be the same for those on different medications, reducing costs and increasing accessibility by having one single intervention which addresses the underlying processes. Successful cognitive behavioural interventions have been developed for specific symptoms such as hot flushes and night sweats (Fenlon et al., 2020; Mann et al., 2012), but it may be that more support for the global burden of ongoing symptoms in survivorship is also important.

### Strengths and limitations

There are several limitations to this current research. Firstly, selection bias may be an issue as people with distress may be less likely to respond to online research advertisements. Equally, it could be that those experiencing symptoms and greater distress were more likely to respond as an opportunity to share their experience. Despite this, there does appear to be variations in the symptoms and distress reported, suggesting a varied sample. As mentioned in the results section, the study was conducted during the COVID‐19 pandemic. Although breast cancer treatment was impacted (e.g., Dave et al., 2021) as reported in this study, feelings of helplessness and being alone were present in participants across the treatment timeline, regardless of whether treatment was initiated before or during the pandemic, indicating a range of experiences not necessarily directly linked to the pandemic. Despite some studies reporting poorer psychological outcomes for those with breast cancer (Swainston et al., 2020) other studies reported little difference in psychological wellbeing compared to before the pandemic (Hulbert‐Williams et al., 2021). The sample only included those who had been on hormone therapy for up to 2 years. Future research could focus on those with longer experience of hormone therapy as medication is prescribed for up to 10 years, to see if these emotional experiences continue. Most women in the sample were of White ethnicity. Hormone receptor positive breast cancer is more common in White women (Cui et al., 2014), so the data could be used to inform future research that may be applicable to these women. However, further research would be useful to focus on breast cancer survivors from other ethnicities to ensure data informing research is applicable and relevant. As recruitment was online, women were recruited from all over the United Kingdom to include different healthcare experiences. A strength of using qualitative interviews is that they add richness and provide in‐depth patient perspectives, which has enabled a deeper understanding of the experiences of hormone therapy that could not be explored in quantitative studies.

## SUMMARY AND CONCLUSIONS

Women on hormone therapy experience distressing physical symptoms. This study contributes to the understanding of *why* these symptoms are distressing for this population of breast cancer survivors. This includes feelings of helplessness around symptoms and managing difficult feelings of loss and change. Understanding and managing the distress related to the side‐effects from taking hormone therapy provides clear targets to improve clinical communication in terms of treatment expectations, validation and acknowledgement and normalizing and compassion, which could help manage some of the uncertainty and worry experienced. Additionally, the data provide information to contribute to intervention development to support these women. Third wave or more traditional cognitive behavioural approaches could be beneficial to help target some of these factors in future interventions. Improving outcomes may in turn have implications for individual and systemic healthcare costs. In addition, there may be implications for patient outcomes both clinically in terms of treatment adherence and recurrence risk, and psychologically in terms of reducing distress and improving quality of life for these women.

## AUTHOR CONTRIBUTIONS

**Sophie Fawson:** Conceptualization; writing – original draft; investigation; project administration; formal analysis; writing – review and editing. **Zoe Moon:** Conceptualization; writing – review and editing. **Melanie Rattue:** Investigation; project administration. **Rona Moss‐Morris:** Conceptualization; writing – review and editing; supervision. **Lyndsay D. Hughes:** Conceptualization; writing – review and editing; supervision; funding acquisition.

## CONFLICT OF INTEREST STATEMENT

The authors declare there are no conflicts of interest.

## Supporting information


Data S1.

## Data Availability

Participants did not consent for data to be shared anonymously in an online repository, so data are not available for sharing.

## References

[bjhp70012-bib-0001] Andreu, Y. , Soto‐Rubio, A. , Ramos‐Campos, M. , Escriche‐Saura, A. , Martínez, M. , & Gavilá, J. (2022). Impact of hormone therapy side effects on health‐related quality of life, distress, and well‐being of breast cancer survivors. Scientific Reports, 12(1), 18673. 10.1038/s41598-022-22971-x 36333362 PMC9636256

[bjhp70012-bib-0002] Ates, O. , Soylu, C. , Babacan, T. , Sarici, F. , Kertmen, N. , Allen, D. , Sever, A. R. , & Altundag, K. (2016). Assessment of psychosocial factors and distress in women having adjuvant endocrine therapy for breast cancer: The relationship among emotional distress and patient and treatment‐related factors. Springerplus, 5(1), 486. 10.1186/s40064-016-2136-2 27218001 PMC4837751

[bjhp70012-bib-0003] Birkhäuer, J. , Gaab, J. , Kossowsky, J. , Hasler, S. , Krummenacher, P. , Werner, C. , & Gerger, H. (2017). Trust in the health care professional and health outcome: A meta‐analysis. PLoS One, 12(2), e0170988. 10.1371/journal.pone.0170988 28170443 PMC5295692

[bjhp70012-bib-0004] Braun, V. , & Clarke, V. (2006). Using thematic analysis in psychology. Qualitative Research in Psychology, 3(2), 77–101. 10.1191/1478088706qp063oa

[bjhp70012-bib-0005] Braun, V. , & Clarke, V. (2019). Reflecting on reflexive thematic analysis. Qualitative Research in Sport, Exercise and Health, 11(4), 589–597. 10.1080/2159676X.2019.1628806

[bjhp70012-bib-0006] Braun, V. , & Clarke, V. (2023). Is thematic analysis used well in health psychology? A critical review of published research, with recommendations for quality practice and reporting. Health Psychology Review, 1–24. 10.1080/2159676X.2019.1704846 36656762

[bjhp70012-bib-0007] Burgess, C. , Cornelius, V. , Love, S. , Graham, J. , Richards, M. , & Ramirez, A. (2005). Depression and anxiety in women with early breast cancer: Five year observational cohort study. BMJ, 330(7493), 702. 10.1136/bmj.38343.670868.d3 15695497 PMC555631

[bjhp70012-bib-0008] Clancy, C. , Lynch, J. , Oconnor, P. , & Dowling, M. (2020). Breast cancer patients' experiences of adherence and persistence to oral endocrine therapy: A qualitative evidence synthesis. European Journal of Oncology Nursing, 44, 101706. 10.1016/j.ejon.2019.101706 32007696

[bjhp70012-bib-0009] Costanzo, E. S. , Lutgendorf, S. K. , Mattes, M. L. , Trehan, S. , Robinson, C. B. , Tewfik, F. , & Roman, S. L. (2007). Adjusting to life after treatment: Distress and quality of life following treatment for breast cancer. British Journal of Cancer, 97(12), 1625–1631. 10.1038/sj.bjc.6604091 18000503 PMC2360272

[bjhp70012-bib-0010] Cui, Y. , Deming‐Halverson, S. L. , Shrubsole, M. J. , Beeghly‐Fadiel, A. , Fair, A. M. , Sanderson, M. , Shu, X.‐O. , Kelley, M. C. , & Zheng, W. (2014). Associations of hormone‐related factors with breast cancer risk according to hormone receptor status among white and African American women. Clinical Breast Cancer, 14(6), 417–425. 10.1016/j.clbc.2014.04.003 24970715 PMC4253543

[bjhp70012-bib-0011] Dave, R. V. , Kim, B. , Courtney, A. , O'Connell, R. , Rattay, T. , Taxiarchi, V. P. , Kirkham, J. J. , Camacho, E. M. , Fairbrother, P. , & Sharma, N. (2021). Breast cancer management pathways during the COVID‐19 pandemic: Outcomes from the UK ‘Alert Level 4’ phase of the B‐MaP‐C study. British Journal of Cancer, 124(11), 1785–1794. 10.1038/s41416-020-01234-4 33767422 PMC7993073

[bjhp70012-bib-0012] DiMatteo, M. R. , & Haskard‐Zolnierek, K. B. (2011). Impact of depression on treatment adherence and survival from cancer. Depression and Cancer, 101–124. 10.1002/9780470972533

[bjhp70012-bib-0013] Early Breast Cancer Trialists' Collaborative Group, E . (2011). Relevance of breast cancer hormone receptors and other factors to the efficacy of adjuvant tamoxifen: Patient‐level meta‐analysis of randomised trials. The Lancet, 378(9793), 771–784. 10.1016/s0140-6736(11)60993-8 PMC3163848 21802721

[bjhp70012-bib-0014] Ebert, D. D. , Van Daele, T. , Nordgreen, T. , Karekla, M. , Compare, A. , Zarbo, C. , Brugnera, A. , Øverland, S. , Trebbi, G. , & Jensen, K. L. (2018). Internet‐and mobile‐based psychological interventions: Applications, efficacy, and potential for improving mental health. European Psychologist, 23, 167–187. 10.1027/1016-9040/a000318

[bjhp70012-bib-0015] Fang, C. Y. , & Schnoll, R. A. (2002). Impact of psychological distress on outcomes in cancer patients. Expert Review of Pharmacoeconomics & Outcomes Research, 2(5), 495–506. 10.1586/14737167.2.5.495 19807473

[bjhp70012-bib-0016] Fawson, S. (2024). Understanding psychosocial factors that are associated with distress and symptom experience in breast cancer survivors on hormone therapy. King's College London. https://kclpure.kcl.ac.uk/portal/en/studentTheses/understanding‐psychosocial‐factors‐that‐are‐associated‐with‐distr

[bjhp70012-bib-0017] Fenlon, D. , Maishman, T. , Day, L. , Nuttall, J. , May, C. , Ellis, M. , Raftery, J. , Turner, L. , Fields, J. , & Griffiths, G. (2020). Effectiveness of nurse‐led group CBT for hot flushes and night sweats in women with breast cancer: Results of the MENOS4 randomised controlled trial. Psycho‐Oncology, 29(10), 1514–1523. 10.1002/pon.5432 32458473 PMC7590063

[bjhp70012-bib-0018] Garreau, J. R. , DeLaMelena, T. , Walts, D. , Karamlou, K. , & Johnson, N. (2006). Side effects of aromatase inhibitors versus tamoxifen: The patients' perspective. The American Journal of Surgery, 192(4), 496–498. 10.1016/j.amjsurg.2006.06.018 16978958

[bjhp70012-bib-0019] Graham, C. D. , Gouick, J. , Krahé, C. , & Gillanders, D. (2016). A systematic review of the use of acceptance and commitment therapy (ACT) in chronic disease and long‐term conditions. Clinical Psychology Review, 46, 46–58. 10.1016/j.cpr.2016.04.009 27176925

[bjhp70012-bib-0020] Hall, L. H. , King, N. V. , Graham, C. D. , Green, S. M. , Barber, A. , Neal, R. D. , Foy, R. , Clark, J. , Lloyd, K. E. , & Smith, S. G. (2022). Strategies to self‐manage side‐effects of adjuvant endocrine therapy among breast cancer survivors: An umbrella review of empirical evidence and clinical guidelines. Journal of Cancer Survivorship, 16, 1296–1338. 10.1007/s11764-021-01114-7 34664199 PMC9630394

[bjhp70012-bib-0021] Harrow, A. , Dryden, R. , McCowan, C. , Radley, A. , Parsons, M. , Thompson, A. M. , & Wells, M. (2014). A hard pill to swallow: A qualitative study of women's experiences of adjuvant endocrine therapy for breast cancer. BMJ Open, 4(6), e005285. 10.1136/bmjopen-2014-005285 PMC4067895 24928595

[bjhp70012-bib-0022] Hayes, S. C. , Luoma, J. B. , Bond, F. W. , Masuda, A. , & Lillis, J. (2006). Acceptance and commitment therapy: Model, processes and outcomes. Behaviour Research and Therapy, 44(1), 1–25. 10.1016/j.brat.2005.06.006 16300724

[bjhp70012-bib-0023] Hoffman, A. J. (2013). Enhancing self‐efficacy for optimized patient outcomes through the theory of symptom self‐management. Cancer Nursing, 36(1), E16–E26. 10.1097/NCC.0b013e31824a730a 22495550 PMC3526102

[bjhp70012-bib-0024] Hulbert‐Williams, N. J. , Leslie, M. , Hulbert‐Williams, L. , Smith, E. , Howells, L. , & Pinato, D. J. (2021). Evaluating the impact of COVID‐19 on supportive care needs, psychological distress and quality of life in UK cancer survivors and their support network. European Journal of Cancer Care, 30(5), e13442. 10.1111/ecc.13442 33764611 PMC8250124

[bjhp70012-bib-0025] Hunter, M. S. , Grunfeld, E. A. , Mittal, S. , Sikka, P. , Ramirez, A.‐J. , Fentiman, I. , & Hamed, H. (2004). Menopausal symptoms in women with breast cancer: Prevalence and treatment preferences. Psycho‐Oncology, 13(11), 769–778. 10.1002/pon.793 15386641

[bjhp70012-bib-0026] Ibrar, M. , Peddie, N. , Agnew, S. , Diserholt, A. , & Fleming, L. (2022). Breast cancer survivors' lived experience of adjuvant hormone therapy: A thematic analysis of medication side effects and their impact on adherence. Frontiers in Psychology, 13, 861198. 10.3389/fpsyg.2022.861198 35602711 PMC9120958

[bjhp70012-bib-0027] Jacobs, J. M. , Walsh, E. A. , Park, E. R. , Berger, J. , Peppercorn, J. , Partridge, A. , Horick, N. , Safren, S. A. , Temel, J. S. , & Greer, J. A. (2020). The patient's voice: Adherence, symptoms, and distress related to adjuvant endocrine therapy after breast cancer. International Journal of Behavioral Medicine, 27, 687–697. 10.1007/s12529-020-09908-2 32495240 PMC7657969

[bjhp70012-bib-0028] Krebs, P. , Prochaska, J. O. , & Rossi, J. S. (2010). A meta‐analysis of computer‐tailored interventions for health behavior change. Preventive Medicine, 51(3), 214–221. 10.1016/j.ypmed.2010.06.004 20558196 PMC2939185

[bjhp70012-bib-0029] Kwan, M. L. , Roh, J. M. , Laurent, C. A. , Lee, J. , Tang, L. , Hershman, D. , Kushi, L. H. , & Yao, S. (2017). Patterns and reasons for switching classes of hormonal therapy among women with early‐stage breast cancer. Cancer Causes & Control, 28(6), 557–562. 10.1007/s10552-017-0888-9 28349440 PMC5439523

[bjhp70012-bib-0030] Lambert, L. K. , Balneaves, L. G. , Howard, A. F. , & Gotay, C. C. (2018). Patient‐reported factors associated with adherence to adjuvant endocrine therapy after breast cancer: An integrative review. Breast Cancer Research and Treatment, 167, 615–633. 10.1007/s10549-017-4561-5 29110151

[bjhp70012-bib-0031] Lethborg, C. E. , Kissane, D. , Burns, W. I. , & Snyder, R. (2000). “Cast Adrift” the experience of completing treatment among women with early stage breast cancer. Journal of Psychosocial Oncology, 18(4), 73–90. 10.1300/J077v18n04_05

[bjhp70012-bib-0032] Malterud, K. , Siersma, V. D. , & Guassora, A. D. (2016). Sample size in qualitative interview studies: Guided by information power. Qualitative Health Research, 26(13), 1753–1760. 10.1177/1049732315617444 26613970

[bjhp70012-bib-0033] Mann, E. , Smith, M. J. , Hellier, J. , Balabanovic, J. A. , Hamed, H. , Grunfeld, E. A. , & Hunter, M. S. (2012). Cognitive behavioural treatment for women who have menopausal symptoms after breast cancer treatment (MENOS 1): A randomised controlled trial. The Lancet Oncology, 13(3), 309–318. 10.1016/S1470-2045(11)70364-3 22340966 PMC3314999

[bjhp70012-bib-0034] Moon, Z. , Hunter, M. S. , Moss‐Morris, R. , & Hughes, L. D. (2016). Factors related to the experience of menopausal symptoms in women prescribed tamoxifen. Journal of Psychosomatic Obstetrics and Gynecology, 38, 1–235. 10.1080/0167482x.2016.1216963 27583832 PMC5556753

[bjhp70012-bib-0035] Moon, Z. , Moss‐Morris, R. , Hunter, M. , Norton, S. , & Hughes, L. (2020). Exploring the needs of women prescribed endocrine therapy from minority ethnic backgrounds. Psycho‐Oncology, 29, 19.

[bjhp70012-bib-0036] Moon, Z. , Moss‐Morris, R. , Hunter, M. S. , & Hughes, L. D. (2017). Understanding tamoxifen adherence in women with breast cancer: A qualitative study. British Journal of Health Psychology, 22(4), 978–997. 10.1111/bjhp.12266 28850763

[bjhp70012-bib-0037] Moon, Z. , Moss‐Morris, R. , Hunter, M. S. , Norton, S. , & Hughes, L. D. (2019). Nonadherence to tamoxifen in breast cancer survivors: A 12 month longitudinal analysis. Health Psychology, 38(10), 888–899. 10.1037/hea0000785 31343218

[bjhp70012-bib-0038] Morales, L. , Neven, P. , Timmerman, D. , Christiaens, M.‐R. , Vergote, I. , Van Limbergen, E. , Carbonez, A. , Van Huffel, S. , Ameye, L. , & Paridaens, R. (2004). Acute effects of tamoxifen and third‐generation aromatase inhibitors on menopausal symptoms of breast cancer patients. Anti‐Cancer Drugs, 15(8), 753–760. 15494636 10.1097/00001813-200409000-00003

[bjhp70012-bib-0039] O'Brien, B. C. , Harris, I. B. , Beckman, T. J. , Reed, D. A. , & Cook, D. A. (2014). Standards for reporting qualitative research: A synthesis of recommendations. Academic Medicine, 89(9), 1245–1251. 10.1097/ACM.0000000000000388 24979285

[bjhp70012-bib-0040] Peddie, N. , Agnew, S. , Crawford, M. , Dixon, D. , MacPherson, I. , & Fleming, L. (2021). The impact of medication side effects on adherence and persistence to hormone therapy in breast cancer survivors: A qualitative systematic review and thematic synthesis. The Breast, 58, 147–159. 10.1016/j.breast.2021.05.005 34049260 PMC8165559

[bjhp70012-bib-0041] Phoosuwan, N. , & Lundberg, P. C. (2022). Psychological distress and health‐related quality of life among women with breast cancer: A descriptive cross‐sectional study. Supportive Care in Cancer, 30, 1–10. 10.1007/s00520-021-06763-z PMC8857009 34950961

[bjhp70012-bib-0042] QSR International Pty Ltd . (2018). NVivo. In Version 12. https://www.qsrinternational.com/nvivo‐qualitative‐data‐analysis‐software/home

[bjhp70012-bib-0043] Rademaker, L. M. , Gal, R. , May, A. M. , Batenburg, M. C. T. , van der Leij, F. , Bijlsma, R. M. , Verkooijen, H. M. , Doeksen, A. , Ernst, M. F. , Evers, D. J. , van der Pol, C. C. , & Monninkhof, E. M. (2025). Side‐effects in women treated with adjuvant endocrine therapy for breast cancer. The Breast, 80, 104416. 10.1016/j.breast.2025.104416 39978152 PMC11880597

[bjhp70012-bib-0044] Rosedale, M. , & Fu, M. R. (2010). Confronting the unexpected: Temporal, situational, and attributive dimensions of distressing symptom experience for breast cancer survivors. Oncology Nursing Forum, 37(1), E28–E33. 10.1188/10.ONF.E28-E33 20044329

[bjhp70012-bib-0045] Schreier, A. M. , Johnson, L. A. , Vohra, N. A. , Muzaffar, M. , & Kyle, B. (2019). Post‐treatment symptoms of pain, anxiety, sleep disturbance, and fatigue in breast cancer survivors. Pain Management Nursing, 20(2), 146–151. 10.1016/j.pmn.2018.09.005 30527856

[bjhp70012-bib-0046] Sim, J. , Saunders, B. , Waterfield, J. , & Kingstone, T. (2018). Can sample size in qualitative research be determined a priori? International Journal of Social Research Methodology, 21(5), 619–634. 10.1080/13645579.2018.1454643

[bjhp70012-bib-0047] Swainston, J. , Chapman, B. , Grunfeld, E. A. , & Derakshan, N. (2020). COVID‐19 lockdown and its adverse impact on psychological health in breast cancer. Frontiers in Psychology, 11, 2033. 10.3389/fpsyg.2020.02033 32982846 PMC7476556

[bjhp70012-bib-0048] Syrowatka, A. , Motulsky, A. , Kurteva, S. , Hanley, J. A. , Dixon, W. G. , Meguerditchian, A. N. , & Tamblyn, R. (2017). Predictors of distress in female breast cancer survivors: A systematic review. Breast Cancer Research and Treatment, 165(2), 229–245. 10.1007/s10549-017-4290-9 28553684 PMC5543195

[bjhp70012-bib-0049] Tompkins, C. , Scanlon, K. , Scott, E. , Ream, E. , Harding, S. , & Armes, J. (2016). Survivorship care and support following treatment for breast cancer: A multi‐ethnic comparative qualitative study of women's experiences. BMC Health Services Research, 16, 1–14. 10.1186/s12913-016-1625-x 27535665 PMC4989374

[bjhp70012-bib-0050] van den Beuken‐van Everdingen, M. H. , Peters, M. L. , de Rijke, J. M. , Schouten, H. C. , van Kleef, M. , & Patijn, J. (2008). Concerns of former breast cancer patients about disease recurrence: A validation and prevalence study. Psycho‐Oncology: Journal of the Psychological, Social and Behavioral Dimensions of Cancer, 17(11), 1137–1145. 10.1002/pon.1340 18484568

[bjhp70012-bib-0051] Waller, A. , Williams, A. , Groff, S. L. , Bultz, B. D. , & Carlson, L. E. (2013). Screening for distress, the sixth vital sign: Examining self‐referral in people with cancer over a one‐year period. Psycho‐Oncology, 22(2), 388–395. 10.1002/pon.2102 22135205

[bjhp70012-bib-0052] Wen, K.‐Y. , Smith, R. , Padmanabhan, A. , & Goldstein, L. (2017). Patient experience of taking adjuvant endocrine therapy for breast cancer: A tough pill to swallow. Patient Experience Journal, 4(3), 104–114. 10.35680/2372-0247.1173

[bjhp70012-bib-0053] Willig, C. (2013). EBOOK: Introducing qualitative research in psychology. McGraw‐Hill Education.

