# Supplementary material for: Exploring physical symptoms and distress in early‐stage breast cancer survivors on hormone therapy: A qualitative study
Source: Br J Health Psychol. 2025 Aug 18;30(3):e70012. doi: 10.1111/bjhp.70012 (PMC12359020; doi:10.1111/bjhp.70012)
Supplement: Supplementary file 1 — Data S1. [file BJHP-30-0-s001.docx]

**Supporting information**

**Contents of supporting information**

Section S1. *Page 1.* Interview schedule

Section S2. *Page 2.* Reflexivity

Table S3. *Page 3.* Physical symptoms reported

Table S4. *Page 4.* Quotes for the overarching theme ‘the emotional burden of symptoms’

Table S5. *Page 5.* Additional quotes for themes

Section S1. Interview schedule

- (rapport builder) So first of all, please could you tell me a bit about your breast cancer diagnosis?

*Prompts if needed:* How long have you been on hormone therapy? Which drug?

- For the next few questions, I am going to ask you about your experiences since you’ve been prescribed hormone therapy.
- Have you experienced distress about your cancer or treatment? If so, can you tell me more about your experience of this? *Use ‘emotions’ if unsure of term distress.*

And how did you or do you manage that distress?

- Have you experienced physical symptoms related to your cancer or treatment? If so, can you tell me about this?

What do you do when you have these symptoms?

And how did you or do you manage those symptoms?

How do you find this? How do you feel when you respond/behave/act like that?

- *If participant indicates link between symptoms and distress, explore this:*

*E.g., You mentioned your symptoms were distressing, can you explain what you mean by that?*

Section S2. Reflexivity

As an insider researcher, I SF have prior knowledge of the experiences of women taking hormone therapy which guided the development of the research question. I made sure to develop a very open interview schedule, that although covered distress, made no assumptions about these experiences. I took an open, curious and interested approach. Having this prior knowledge and understanding helped to create a good rapport with the participants, although with respect to the lived experience I am very much an outsider as I do not have personal experience of breast cancer or hormone therapy. The insider knowledge allowed participants to feel comfortable and open with their discussions and it meant the participants knew they didn’t have to explain concepts in too much detail and rather respond to the questions asked. I am also female and think that helped with discussions of more intimate topics. Having two independent interviewers may have introduced some bias, however, the other interviewer, a BSc student, had a complimentary approach, as even though they were less familiar with the area, they received extensive interview training and used conversational prompts to help build rapport. Building rapport with participants was important to enable truthful and non-judgemental discussion of participants’ lives and allowed the participant to lead the conversation.

Table S3. *Physical symptoms reported during the interviews*

| Side effect/symptom reported | *n* |
| --- | --- |
| Joint pain, aches, bone pain | 15 |
| Hot flushes and/or night sweats | 14 |
| Insomnia and/or sleep difficulties | 10 |
| Fatigue and/or brain fog | 10 |
| Weight gain | 4 |
| Vaginal/sex issues | 4 |
| Headaches or migraines | 4 |
| Nausea on zoladex injection | 1 |
| Dizziness | 1 |
| High blood pressure | 1 |
| Bladder urgency | 1 |

| Table S4. *The emotional burden of symptoms quotes* |
| --- |
| *‘I think* ***annoyance and frustration****, because even on the tamoxifen I do find I have a lot of joint pain which I didn't have before.’ P20, 75, anastrozole then tamoxifen*  *‘It’s (sweats) horrible, it’s really* ***horrible*** *[…] When the hot sweats come. I think that’s when, you know you think to yourself oh my God and then the* ***anger****, sometimes* ***naggy****. Really naggy, you know, and I'm like, oh why is it me and I’m sitting there sweating.’ P15, 51, tamoxifen*  *‘I mean I was relatively fit so it's you know, kind of it shouldn't have ached that much, you know. But yeah it, it was* ***horrid****.’ P7, 64, anastrozole then exemestane*  *‘And then you're obviously put into this accelerated menopause. So that was my* ***hardest*** *bit.’ P1, 46, tamoxifen*  *‘Pretty, pretty* ***rotten*** *really.’ P12, 49, letrozole and goserelin*  *‘So I'd say those are the* ***distressing*** *parts that the symptoms that come with taking tamoxifen is what really can take you down. I went through a period of really* ***low mood****.’ P8, 49, tamoxifen*  *‘Now, whether it was the hormone therapy's impact on my mood, or whether it was just the fact that you know my hands were getting stiffer and stiffer and stiffer and clickier by the day that was having an impact on my mood, I don't know […] But what I do find quite* ***galling*** *is it is the pain in the stiffness in the joints that is really quite um, can be quite acute at times.’ P16, 47, exemestane*  *‘I think I put on about half stone. Um? Since I started taking these hormone blockers, um, I'm not a very tall person. I’m only 5 foot two I think I put on half a stone when I started the menopause and then another half a stone and I thought this is, you know, this is* ***horrendous*** *[…]* *Look putting the weight on is really* ***stressful****. Even though I didn't have chemotherapy, your hair thins, and all of the side effects are really* ***stressful****.’ P11, 53, anastrozole then letrozole*  *‘I had, you know I think having the sleep disruption is it's really* ***not fun****.’ P13, 51, tamoxifen*  *‘And the side effects are very very* ***stressful****. And I think they make you feel quite* ***depressed*** *because it just becomes* ***self-consuming*** *if you just think about them all the time.’ P11, 53, anastrozole then letrozole* |

*Note: words related to distress/emotion are in bold font*

Table S5. *Additional quotes*

| Theme | Additional quotes |
| --- | --- |
| The emotional burden of symptoms (overarching theme) | *‘Sometimes I think the mental side effects are even bigger than the physical side effects.’ P11, 53, anastrozole then letrozole*  *‘aware that for many people they have a much greater impact’ P23, 49, letrozole*  *‘I'm not weighed down by it every day but my cancer, you know but I still do have symptoms, I do still have side effects so it's it is still a very lived experience.’ P22, 49, tamoxifen*  *‘Well at my age, I don't really worry much about it.’ P20, 75, anastrozole then tamoxifen* |
| 1. A sense of helplessness around symptoms | *1.1*  *I think people assume that if you look normal that everything feels normal […] just because I’ve finished chemo and my hair’s grown back doesn’t mean to say that everything’s a bed of roses. P21, 54, anastrozole*  *‘I told the oncologist this, thinking she would go oh yeah, well that just happens, some people get weird sensations in their bones, but I'm not liking my oncologist. She went, what do you want me to do?’ P1, 46, tamoxifen*  *‘I was actually astounded that there was no support […] went up to this kind of handover meeting in the (hospital name) and they gave me a Suppository tube which I thought why don't they just hand it over to everyone who's on tamoxifen? […] thought this is unnecessarily difficult.’ P18, 48, tamoxifen*  *And I've been told by a few* [oncologists], *oh, it's not the tamoxifen, it was the chemo and I'm like, well, what about the women who didn't have chemo who were taking tamoxifen and are experiencing really bad weight gain? […] All honesty, in the medical world, the doctors, the GPs, the oncologist. I don't think they really give much um, what's the word? I don't think they really take on board fully. Um, how it impacts a person*. *P8, 49, tamoxifen*  *[They] reassured me that these things [side effects] can go on; there isn't a timeline for them […] knowing that it’s normal […] just knowing there was nothing unexpected going on, you know, just for me gives me the ability to kind of go OK, it is what it is. P22, 49, tamoxifen*  *And then when I mentioned it to the, our I think breast cancer nurse, it could have been at one point, and I said about the joint pain and she goes “well that’a unusual ‘cause usually you don’t get joint pain, with tamoxifen”. I thought well obviously you don’t read that group (tamoxifen Facebook group) that I go on because there’s they have had everything and everything you know, so […] ‘Cause the doctor, thinks the symptoms could be menopause related. And I’m like, well I know they’re not because you go on the website and tamoxifen says it's got all them symptoms so.’ P15, 51, tamoxifen*  *‘I'm managing okay. I think a lot of people probably wouldn't know there was anything wrong with me. But I'm aware every time I'm walking, you know that I'm in pain.’ P20, 75, anastrozole then tamoxifen*  *I don't know if it's because I had treatment, it happened during the pandemic […] so I don't know if my experience is the same for all people […] Like having a breast cancer nurse ring me up and talk me through stuff and kind of what helps available. I think would have been better than just, they just kind of chuck the pills at you and that’s it. P1, 46, tamoxifen*  *1.2*  *‘But yeah, the tiredness. I don't. Yeah, haven't really kind of cracked that one. And when I've asked people for suggestions, they’ve been like well there's not nothing you can really do for that, so.’ P13, 51, tamoxifen*  *What you're doing is only coping, you cannot actually make it better or make it worse, but you can cope better with that kind of thing. P18, 48, tamoxifen*  *‘How else do I manage them? Physically, I can't really, I just have to, you just have to get on with it […] that's one of the difficulties with the hot flashes and stuff is there's nothing you really can do. You just gotta, you know, just gotta ride it out and like I say I'm trying Acupuncture hasn't really done anything yet.’ P19, 43, tamoxifen and goserelin*  *‘And, but, and I'm not really aware of anything else, I mean the migraines, the doctors giving me some atriprarim to combat them, and it does help when I have them, but I'm I'm always a bit of the warning leaflet the info leaflet says if you use them too often it can make them worse so I try not to use it too often. But you know, when I get a migraine, it's the only thing that'll touch it paracetamol, ibuprofen just do nothing.’ P12, 49, letrozole and goserelin*  *It's like you're taking a tablet to take a tablet. P15, 51, tamoxifen*  *Because she [oncologist] had said well I can refer you to a surgeon [for] carpal tunnel syndrome. I said I don't really want to go down that avenue. I can't go for more surgery on what this, what the letrozole was causing. P4, 57, letrozole, exemestane then tamoxifen*  *‘What helped with the distress level that I had there when I was thinking of coming off it was, I actually felt like I had more information, more choice and more control […] I know what my options are and I go OK, but I'm sticking with it […] I know that if I get to January and things are still intolerable, I can do something about it and I think having that sense of agency is really important […] You know that there is actually an escape clause.’ P16, 47, exemestane*  *‘Once all my treatment options are over and done with. I don’t want to start throwing more drugs down my neck unless I've got too.’ P9, 63, anastrozole* |
| 2. Difficult feelings around loss and change | *‘Certainly I think, um, menopause brain. If I can call it that combined with chemo brain is literally doing my nut in because I'm um, I've always had a very good memory. I've always had a really good memory, really articulate, and now in the middle of sentences I'm like “errr” I hate that God I hate that.’ P16, 47, exemestane*  *‘But the headaches do. But more. More cause I suppose in a way it sort of spoils things 'cause you know, like if you've got plans, then you've got a really bad headache and you can't go somewhere or do something, like I can't drive when I get them.’ P17, 44, anastrozole*  *Then you start feeling quite achy and lethargic and fatigued and quite depressed as well. And I think it makes your body less resilient to things that you might have ordinarily think oh, I can do that really easily, and all of a sudden you can't, so that from a mental perspective.* *P11, 53, anastrozole then letrozole*  *But then other days it really gets me down because without getting too personal, like having sex and stuff like, you don't, you know, a vaginal dryness for want of a better word, that is something I've never experienced and it's really difficult because that has always been an important part of my life. P19, 43, tamoxifen, goserelin*  *‘I have definitely got it has affected me and this is where the hormone therapy, whether it's chemo or hormone therapy, that definitely an element of hormone therapy to it and a big element has affected my life and what I can do […] but the fatigue has, affected how much I can do […] no absolutely treatment and as part of that is hormone therapy has affected my lifestyle.’ P23, 49, letrozole*  *‘I think my age, 'cause I'm still quite young, I'm so yeah, I was 44 when I was diagnosed and I’m 45 now I do feel having to go through the menopause quite early, it that's a bit, so that makes you bit, not depressed, but I think I could have had another 10 years before my body starts to deteriorate.’ P14, 45, anastrozole and goserelin*  *‘It's the side effects that have been really hard to deal with, and I'm someone who is really fit. Very active. I'm only 53. It's a very hard drug to take.’ (P11, 53, anastrozole then letrozole)*  *Yeah, I just think because I've been someone who's never had to take tablets or never had any health issues all of a sudden being on daily medication, monthly medication and knowing that that is the way it has to be for 10 years. Is yeah, it’s not very nice. P19, 43, tamoxifen and goserelin*  *I should be feeling better now, so it's a kind of different, it's a different worry if you know what I mean. It's like, am I ever gonna get back to normal again? P19, 43, tamoxifen and goserelin*  *‘Quite down because you know it. I like to go out and about and do lots of bits and you didn't feel quite the same as you know, going out so much and I guess when you're in pain you get a little bit snappier and all of those things and it, it's just not how I like to be, if that makes sense.’ P7, 64, anastrozole then exemestane*  *‘realise I can just focus on what I want to do another day. If I'm not able to do it that day […] It's just being something I've had to deal with and get through and deal with the loss of the things I can't do anymore.’ P14, 45, anastrozole and goserelin* |
| 3. Living with uncertainty around side effects | *‘I read up a lot about tamoxifen and all the side effects, so I was really apprehensive actually before taking it’ P5, 39, tamoxifen*  *‘I think the one that is completely unsaid out of all of the side effects that I actually had no idea is the sexual side of things. The sexual side effect. And that really is an area that is not, not. You just unaware, I had no idea. I had no idea of the impact and that is a big impact. A severe you know.’ P23, 49, letrozole*  *‘With specifically in regards to hormone therapy, yeah, it was very, very unexpected. I think the most difficult thing was it was unexpected […] and I thought that's going to be the easy bit because I'm 47. A lot of my friends have already gone through the menopause.’ P18, 48, tamoxifen*  *‘Then obviously when you go to bed as much as I enjoy lying in the bed, stretching my body out, feeling myself sinking off, but then get that hot flash turn the fan on, it's just like. Almost like Groundhog Day, you know? Chucking off the covers and it's just like when is this gonna stop? I don't know, that's the frustrating part really.’ P8, 49, tamoxifen*  *‘And then forgetfulness […] Now, last night I left the lid off. It's not a huge catastrophe, but then you sort of look at the other areas of your life and go. Where else is this, you know? Am I going to get into the car one day and forget, you know mirror signal manoeuvre, or you know something like that. So yes, leaving the lid off the dog food, not a big thing. Potentially it could have broader implications.’ P16, 47, exemestane*  *‘Rather than getting in touch with the hospital all the time and asking them is, is it normal? What should be happening? Is there something wrong with me?’ P14, 45, anastrozole and goserelin*  *‘But apparently that's normal. I've been led to believe so it's kind of I just and it's just you, you know that sort of your body is not the same as it was, which is something to reconcile with.’ P13, 51, tamoxifen*  *‘So I understand that the situation is what it is, I’m struggling with my hips, it’s a normal side effect.’ P21, 54, anastrozole*  *‘Fingers crossed, but it is, it is early days for me.’ P6, 56, tamoxifen*  *‘And then I think the fear of coming off it and going on the other ones is even worse ‘cause they say that causes joint pain.’ P15, 51, tamoxifen*  *‘I'm hoping really, soon when the menopause maybe calms down a little bit, you know. Maybe that's when you know it may subside a bit.’ P15, 51, tamoxifen*  *It's a little bit harder because it's more like oh you're going to be on this for 10 years. You know it's like, oh God, is this, is this my life? […] The hot flashes, headaches […] I've gotta be on these for 10 years. It, you can't see an end to it. […] is this what it's gonna be like now.’ P19, 43, tamoxifen and goserelin* |
| 4. The internal conflict around treatment decisions | *‘…obviously it was quality life as well. You can't spend the rest of your life 10 years absolutely racked in pain.’ P4, 57, letrozole, exemestane, tamoxifen*  ‘*It's kind of between a rock and a hard place sometimes.’ P1, 46, tamoxifen*  *‘It's very difficult to make an informed choice, even as I said, if you're very educated and you start to understand the questions to ask, for me it's the upfront discussion [yeah]. You just don't know what to ask because I don't think you actually hear what people are telling you. It is just a blur.’ P11, 53, anastrozole then Letrozole*  *‘So if there's two in every hundred people that possibly coming back, do you really need to take the tablet? It's like is it really needed for everybody you know, as in everything that they give to you.’ P15, 51, tamoxifen*  *‘So I did notice patterns initially that when I missed the tamoxifen, there was it, I've kind of found my old me when I got up and I was surprised that it was just for a day, because, you know, even for a day, you wouldn't because it’s a long term medication. You wouldn't think that we missed one. It's not, it shouldn't affect you that much.’ P18, 48, tamoxifen*  *‘And when I had a six weeks holiday from the drugs, I did lose half a stone which felt really, really good. […] but now I'm back on the drugs. It's getting really hard even to lose like half not even half a pound.’ P11, 53, anastrozole then Letrozole*  *‘I’m not perfect and I've got the aches and pains of letrozole … but I’m still around from a kids.’ P2, 63, letrozole*  *‘But you kind of say to yourself if that's the cost of not being one of those two people* (who might get a recurrence)*.’ P11, 53, anastrozole then letrozole*  *Yeah, Tamoxifen is tough in that, you know you have to take it because obviously it's what's helping to stave off the oestrogen.’ P8, 49, tamoxifen*  *I need to do that for myself and I need to do that for my family and friends.’ P3, 33, tamoxifen*  *I want to see my kids grow up and that sort of thing I don't want it to come back. P12, 49, letrozole, goserelin*  *‘I'm kind of more grateful that the tamoxifen gives me kind of hope more than anything in the way.’P5, 39, tamoxifen*  *And if it's a few side effects, but I'm here, then it don't matter. I'll cope with them.’ P17, 44, anastrozole* |
